# Using Behavioural Insights to Promote Food Waste Recycling in Urban Households—Evidence From a Longitudinal Field Experiment

**DOI:** 10.3389/fpsyg.2018.00352

**Published:** 2018-03-22

**Authors:** Noah Linder, Therese Lindahl, Sara Borgström

**Affiliations:** ^1^Environmental Psychology, Department of Building, Energy, and Environmental Engineering, University of Gävle, Gävle, Sweden; ^2^The Beijer Institute of Ecological Economics, Royal Swedish Academy of Sciences, Stockholm, Sweden; ^3^Stockholm Resilience Centre, Stockholm University, Stockholm, Sweden; ^4^Sustainable Development, Environmental Science and Engineering, Royal Institute of Technology, Stockholm, Sweden

**Keywords:** pro-environmental behaviour, nudging, community-based social marketing, food waste recycling, natural field experiment, longitudinal, difference-in-difference analysis

## Abstract

Promoting pro-environmental behaviour amongst urban dwellers is one of today's greatest sustainability challenges. The aim of this study is to test whether an information intervention, designed based on theories from environmental psychology and behavioural economics, can be effective in promoting recycling of food waste in an urban area. To this end we developed and evaluated an information leaflet, mainly guided by insights from nudging and community-based social marketing. The effect of the intervention was estimated through a natural field experiment in Hökarängen, a suburb of Stockholm city, Sweden, and was evaluated using a difference-in-difference analysis. The results indicate a statistically significant increase in food waste recycled compared to a control group in the research area. The data analysed was on the weight of food waste collected from sorting stations in the research area, and the collection period stretched for almost 2 years, allowing us to study the short- and long term effects of the intervention. Although the immediate positive effect of the leaflet seems to have attenuated over time, results show that there was a significant difference between the control and the treatment group, even 8 months after the leaflet was distributed. Insights from this study can be used to guide development of similar pro-environmental behaviour interventions for other urban areas in Sweden and abroad, improving chances of reaching environmental policy goals.

## Introduction

Most sustainability problems we face today (e.g., global warming, biodiversity loss, deforestation, water and air pollutions, and overfishing) are rooted in human behaviour (Vlek and Steg, 2007). Behaviour among urban dwellers stand for a disproportionally large share of global resource use (Grimm et al., 2008), which is predicted to increase even further, as the number of urban citizens is predicted to grow from 3.2 billion (2005), to ~6.4 billion by 2050 (UN, 2014, 2015; UN-Habitat, 2016). Thus, to avoid potentially catastrophic global environmental change, promoting pro-environmental behaviour amongst urban inhabitants needs to be a top priority, both for policy and for research (Brewer and Stern, 2005; Vlek and Steg, 2007; Clayton et al., 2016).

Even seemingly small behaviour changes can have a big aggregate impact. One estimate suggests that an emission reduction of 123 million tons of carbon dioxide per year over 10 years (7.4% of US national emissions) can be achieved by relatively small alterations in behaviour (e.g., switching to low-flow showerheads, efficient water heaters or more fuel-efficient vehicles) in United States households and amongst non-business travellers (Dietz et al., 2009). One household level behaviour with potentially large positive impacts is recycling of food waste. Out of all the food produced in the world approximately one third is lost or wasted (Gustavsson et al., 2011). Food loss and waste stand for 8% of global greenhouse gas emissions, consume a quarter of all water used by agriculture, and generate about $940 billion in economic losses globally (FAO, 2015). There is no doubt a big potential in changing the way we manage our food, where a reduction in food wasted is a key element in creating a sustainable food system. Simply reducing food waste would naturally be the most effective tool (Lipinski et al., 2013) but around 20% of all household food waste is unavoidable, e.g., peels, shells, and bones, (Quested et al., 2011) and recycling unavoidable food waste has clear societal benefits, such as reducing associated greenhouse gases and pollution from landfills, creating bio-gas to replace fossil fuels, and bio-fertilizer (digestate) that can recycle nutrients and organic matter back to the soils (Al Seadi et al., 2013). Sweden (alongside other countries) has recently set strong policy goals to develop organic collection programs intended to increase the amount of food waste recycled from 38% of the total food waste in 2014 to 50% in 2018, which equals an annual increase of 112 200 tons of food waste (Naturvårdsverket, 2017). To achieve such goals large scale behaviour changes are needed, but organic collection programs developed today tend to focus on structural changes and technological development, often overlooking the essential role that households' behaviours play in reaching these goals (Geislar, 2017).

Changing human behaviour is seldom a straightforward process. Information based campaigns are commonly used to promote behaviour changes, their goal is often to alter attitudes or enhance knowledge about an environmental problem and in that way promote behaviour changes. Unfortunately this attitude-behaviour approach is often an ineffective way of sparking behaviour change, and when evaluated, they repeatedly fall short of achieving their goal (McKenzie-Mohr, 2013). Large scale information and advertising campaigns also tend to be very expensive In one rather extreme example, utility companies in American state of California spent yearly about 200-million-dollar on advertising campaigns promoting the installation of energy-efficient devices in households, along with suggestions on behaviour changes that could save energy (like closing windows on sunny days; Archer et al., 1983). However, when evaluated only mixed results could be shown, at best, and audits suggested that there is a weak linkage between consumers receiving conservation information and actually acting on that information (Coltrane et al., 1986).

Scholars in environmental psychology and behavioural economics have long highlighted the fact that insights from behavioural sciences is usually not utilized in the design of campaigns and information strategies trying to promote environmentally friendly behaviour (e.g., McKenzie-Mohr, 2000a,b; Thaler and Sunstein, 2008; Kazdin, 2009). Even though we are constantly surrounded with these messages it is clear that many of them still do not take full advantage of current scientific knowledge. Furthermore, information campaigns are seldom evaluated, at least not with a solid experimental design with one or more “treatment” groups and a comparable control group, analysing “objective” outcome measures e.g., waste weight or kWh (Clayton et al., 2016). Even rarer are long-term evaluations; only a few studies have investigated how treatment effects have lasted or changed over time, stretching more than a couple of weeks past intervention (Osbaldiston and Schott, 2012; Allcott and Rogers, 2014; Anderson et al., 2017). As a result, there is currently little knowledge about how such interventions influence behaviour over time (Clayton et al., 2016).

This study aims to shed some light on the research gaps presented above. In particular we want to explore if insights from environmental psychology and behavioural economics applied in the design process of an information leaflet can increase food waste recycling. We chose an information intervention as it is the standard tool used by housing companies, waste companies, and by municipalities in their efforts to promote recycling behaviour. It is also cheap to implement and easy to scale up or adapt. We designed a natural field experiment (NFE) to test our main hypothesis: The information leaflet increases the amount of food waste collected from sorting stations in our research area (compared to a control group without the intervention). Since food waste is being sorted out from the regular unsorted household waste we also test the following secondary hypothesis: The information leaflet decreases the amount of household waste collected from the sorting stations. Moreover, we are interested in evaluating the potential long-term effects of the leaflet and not only the immediate effects. We observe how behaviour changes over the 8 months following the intervention. This study is to the best of our knowledge the first study analysing an “objective” outcome measure—the actual weight of food waste collected (not self-reported or self-assessed), in a longitudinal study. Even though interventions aimed at reducing food waste have gotten some attention in the behavioural science literature (e.g., Kallbekken and Sælen, 2013; Graham-Rowe et al., 2014; Visschers et al., 2016) there are only a handful of studies focusing on developing and evaluating interventions promoting the sorting and *recycling* of food waste (see e.g., Karim Ghani et al., 2013; Bernstad, 2014; Geislar, 2017).

## Study area

Our study area was Hökarängen, a city district in southern Stockholm (Sweden). At the time of the study around 9,400 people lived in Hökarängen and population data showed a strong political support for the more socialistic leaning political parties, a slightly below average income and enrolment in education above high school (see Table 1).

**Table 1:** Demographic data for Hökarängen compared to the Stockholm (municipality) average, the data was received from Statistik om Stockholm (n.d), and collected in year 2016.

**Demographic data**	**Hökarängen**	**Stockholm average**
Unemployment rate	3.6%	2.8%
Mean income (16 years and over)	255,100 SEK	352,000 SEK
Higher education	42.1%	57.5%
Foreign background (not born in Sweden)	34.6%	32.1%
Politic votes in 2014 election. (Socialistic block)	68.7%	49.8%
Politic votes in 2014 election. (Liberal/conservative block)	19.7%	43.5%

The study was conducted in collaboration with the housing company Stockholmshem, which is owned by the municipality and accommodating more than 50,000 residents (about 5% of the total population of Stockholm municipality). Stockholmhem is Stockholm's largest housing company, and owns around 2,900 of the total 4,700 apartments in Hökarängen.

**Figure 1:**
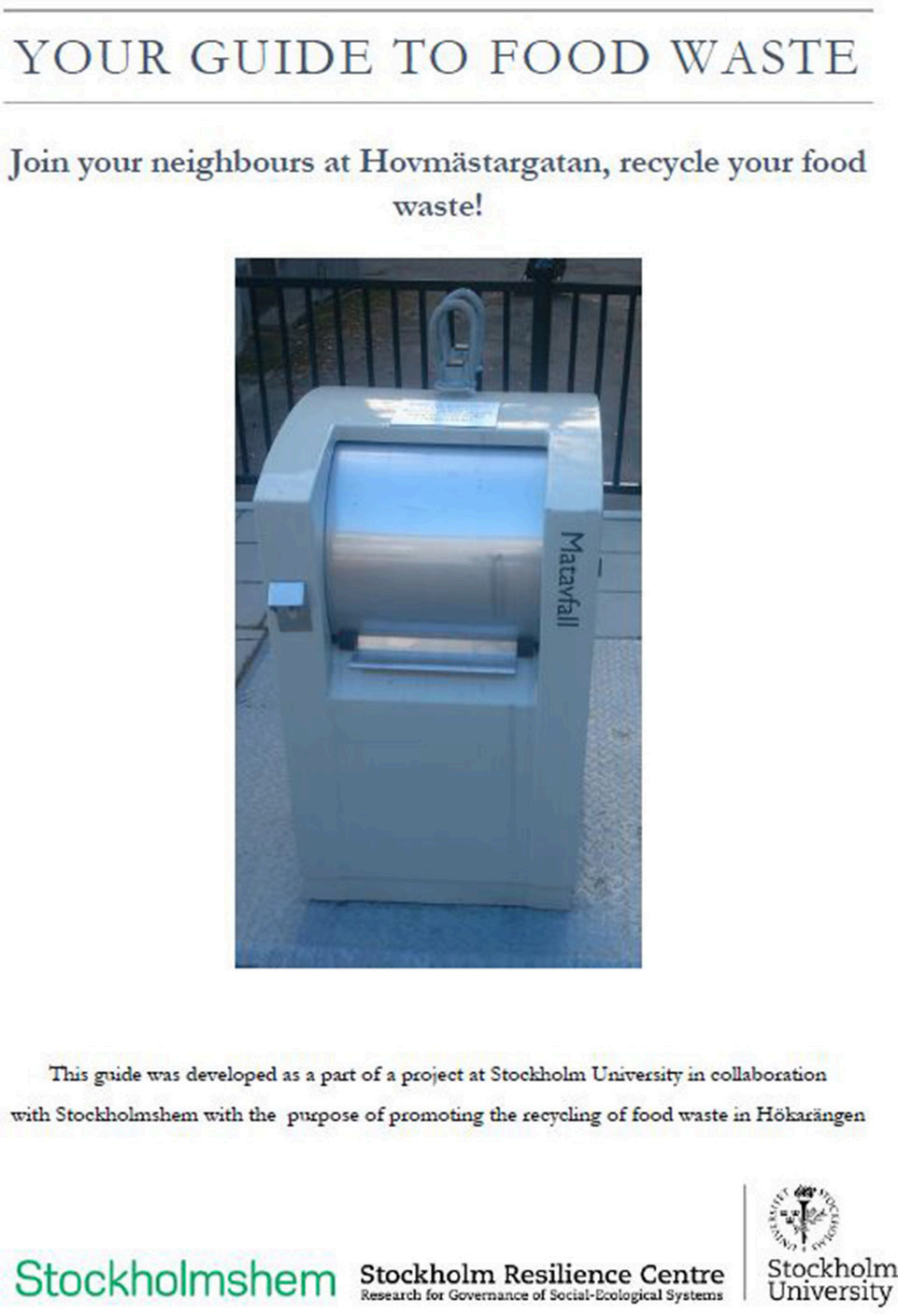
The front page of the information leaflet (translated from Swedish, see the full leaflet Appendix B in Supplementary Material) the picture used was taken by one of the authors.

The majority of the households in Hökarängen have not had the possibility to recycle their food waste. However, in 2014 Stockholmshem started a project to provide their residents with stationary sorting stations outside their apartment complexes for recycling food waste (a picture of a sorting station can be seen in Figure 1) in line with Swedish policy goals. The plan is to eventually install sorting stations for all their apartments in Hökarängen. The research took place in an area where 474 households (about 10 % of all households in Hökarängen) live in area-typical apartment complex consisting of mainly 2 or 3 room apartments, all in six storey buildings (12 apartments in each). All households in the research area had access to food waste sorting stations, and they were installed more than one year before the information leaflet was sent out (except for two stations that were installed 7 months before the intervention, these were divided up into the control- and the treatment group). At the time of the installation Stockholmshem provided their residents with information about the new sorting stations and the possibility to now sort and recycle food waste (all household in the area got the same information when a sorting station was installed). Still, only a few of the households started sorting and recycling food waste, and the desire from Stockholmshem to increase food waste recycling in the area is in part what lead to this collaboration.

## Theoretical background

When designing the information leaflet, we focused primarily on theories from environmental psychology and behavioural economics with the explicit goal to promote pro-environmental behaviour changes. We are using the broad definition of pro-environmental behaviour as: “any human behaviour that either benefits the environment, or harms it as little as possible” (Steg and Vlek, 2009).

A grand challenge for environmental psychologists today is to study, explain, and predict how to promote pro-environmental behaviour (Sörqvist, 2016). Environmental psychology is a branch of psychology science that deals with the complex relations between people and the natural or built environment. In an integrated review of the pro-environmental behaviour research within environmental psychology four key phases are identified as recurring suggestions for how to promote pro-environmental behaviour effectively: (1) identification of the behaviour to be changed, (2) examination of the main factors underlying this behaviour, (3) design and application of implementation to change behaviour (4) evaluation of the effects of implementation (Steg and Vlek, 2009)

There have been numerous studies within psychology research on how and why information interventions succeed or fail to spark pro-environmental behaviour. Even though they often are criticized for being ineffective tools, there are several studies showing positive results from using information interventions (Farrow et al., 2017). Many of these interventions are using messages crafted around social norms, both descriptive norms (the perceptions of which behaviours are typically performed) and injunctive norms (the perceptions of which behaviours are typically approved or disapproved of) are commonly used (Cialdini, 2003). Descriptive norms have shown to be especially effective in some cases, one study aiming to reduce energy consumption showed that using the descriptive norm, of “Join your neighbours in conserving energy” was more effective than the more commonly used injunctive normative message of environmental protection “Protect the environment by conserving energy” or even messages of self-interest “Save money by conserving energy” (Nolan et al., 2008). Similar results have been found when trying to promote hotel guests to reuse towels, and the more specific and “local” the descriptive norm, the more effective it was, e.g., a message along the line with “The guests in this room tend to reuse the towel,” worked better than the more general “The guests in this hotel tend to reuse their towel” (Goldstein et al., 2008). Other pro-environmental behaviours that have been successfully promoted by the use of norms include: recycling (Andersson and von Borgstede, 2010) reducing plastic bag use (De Groot et al., 2013) and water conservation (Bernedo et al., 2014) to name a few. On the other hand, some studies have shown that misusing norms and descriptive norms in particular can generate unwanted backlash effects, even increasing the behaviour the intervention was designed to prevent (Cialdini, 2003).

One of the most prominent theories in promoting pro-environmental behaviour within environmental psychology is community-based social marketing (CBSM) (Schultz, 2014). CBSM has its roots in social marketing which was first coined by Kotler and Zaltman (1971) and presents a framework for incorporating traditional commercial marketing techniques and insights (e.g., planning, pricing, communication, distribution, and marketing) to design more effective social campaigns. Social marketing seeks to influence behaviours that benefit individuals and communities for the greater social good (Lefebvre, 2016). CBSM was initially presented as guidelines on how to make psychological knowledge visible to better develop and deliver programs that promote pro-environmental behaviours by McKenzie-Mohr (2000a,b). It has been tested and used with promising results to promote pro-environmental behaviours such as: inspiring residents to start back-yard composting, reducing travel by car, and increasing curb side recycling rates (McKenzie-Mohr, 2000a,b; Haldeman and Turner, 2009). It has also been successful in delivering programmes addressing several human health issues (Athey et al., 2012). CBSM is aligning with the steps mentioned above by Steg and Vlek (2009) and presents five steps to promote behavioural changes: (1) selecting behaviour (2) identifying barriers and benefits (3) developing strategies (4) piloting, and (5) broad-scale implementation, highlighting the importance of evaluating interventions and adapting them to the specific context (McKenzie-Mohr, 2013). This study will in part follow these steps when designing the information leaflet (see Methodology).

The field of behavioural economics incorporates insights from other social sciences, most notably psychology, in order to enrich the standard economic model by identifying how human behaviour deviates from the assumptions of Homo Economicus. In essence these assumptions suggest that people have well-defined preferences, unbiased beliefs, and that they make optimal choices based on these beliefs and preferences. This in turn implies that people have perfect cognitive abilities and infinite willpower. It is also often assumed that their primary motivation is self-interest (Thaler, 2016). Behavioural economists argue that human behaviour needs to be understood in the light of people having limited cognitive capacities, imperfect willpower and bounded self-interest, all deviations from the Homo Economicus model that have been replicated in numerous studies (see e.g., Kahneman, 2003). Being aware of such deviations, e.g., how heuristics (cognitive rules of thumb), framing information, loss aversion, social pressure etc., influence choices, and knowing when they are more or less present could potentially improve policy design and interventions, which is what Thaler and Sunstein (2008) argue in their book “Nudge”—Improving Decisions about Health Wealth and Happiness.

According to Thaler and Sunstein's (2008) definition, a nudge-intervention is changing some aspect of the decision-context in a way that steer people's behaviour in a predictable direction. To be called a nudge the intervention is not allowed to prohibit or remove any choice alternatives and it must respect people's free will. Furthermore, a nudge is not allowed to drastically change or add any financial incentives. Nudging is mostly used to promote or change a certain behaviour (often without increasing knowledge or changing attitudes). Nudging as a concept has increased immensely in popularity over the last decade, not only among academics, but also among policy makers and in civil society (as exemplified by numerous governmental and non-governmental initiatives with the purpose of applying behavioural insights in policy^1^. Nudging approaches have been successfully applied in many different areas and to different types of behaviour e.g., to increase private savings and tax compliance (see e.g., Hallsworth et al., 2014), reduce energy consumption (Allcott and Mullainathan, 2010; Allcott, 2011; Costa and Kahn, 2013; Allcott and Rogers, 2014), limit water and paper consumption (Egebark and Ekström, 2016), and reduce food waste from restaurants (Kallbekken and Sælen, 2013).

Outside academia nudging is often portrayed as a success story, yet it is a fairly new branch of research and has been heavily critiqued by some scholars. For one, it is seemingly hard to define and distinguish “nudges” from other types of psychology-based interventions and tools and therefore some argue that the term nudge is just a rebranding of already established psychological terms (Kosters and Van der Heijden, 2015). It has also been critiqued for being manipulative (see e.g., Marteau, 2011; Goodwin, 2012) or serving as an easy way out for policy makers that favour them over “harder” policy instruments, such as regulations and economic incentives, when such policy tools in fact are needed (Bonell et al., 2011). Furthermore, the effectiveness of nudging as a public policy tool has been questioned based on the lack of evaluations (Kosters and Van der Heijden, 2015). This latter critique was addressed recently by Benartzi et al. (2017) showing how public nudge interventions can be both cost effective and in some circumstances a favourable option over traditional policy tools.

The nudging literature sometimes suggests similar phases (see e.g., Ly et al., 2013) and often rests on the same psychological insights as CBSM. It does not generally take an equally holistic approach though, focusing mostly on evaluating and quantitatively measuring the effect of a specific tool and on adapting the chosen tool to different decision contexts (Mont et al., 2014; Lindahl and Stikvoort, 2015). Despite the seemingly interlinked research using similar behavioural insight tools surprisingly little cross-referencing exists between CBSM and nudging. In this study we use and combine insights from both these research fields.

## Methodology

### Designing the intervention

The design of the information leaflet was highly influenced by the phases presented above, we combined the frameworks found within environmental psychology (Steg and Vlek, 2009; McKenzie-Mohr, 2013), and included insights from behavioural economics and nudging (e.g., from Sunstein, 2014).^2^ The methodical framework used is presented below:

1. **Selecting Behaviour to Change**
  For this study the choice was made together with Stockholmshem to focus on promoting the pro-environmental behaviour of recycling food waste.
2. **Examination of the Main Factors Underlying the Behaviour**
  To examine the main factors underlying the behaviour, two steps are typically recommended as was also implemented for this study; a pilot study, and a review of literature. The main goal of a pilot study is to learn about the area and to uncover context specific barriers (and benefits) for the desired behaviour, this is often argued to be a crucial step if any form of sustainable behaviour is to be widely adopted (McKenzie-Mohr, 2013). Barriers can be both internal: individual motivation like moral concerns or normative influences, and external: barriers that varies with community, e.g., accessibility, convenience, or cost of changing the behaviour (Steg and Vlek, 2009), and barrier removal is often at the core of successful interventions (Lorenzoni et al., 2007). The main goal of the pilot study in Hökarängen was to identify barriers for the residents to recycle food waste, to get an overview of their attitudes toward sorting food waste, and to get a rough estimate of the residents already recycling food waste. To this end, the pilot study was carried out in two phases, where the first phase was visiting and learning about the research area, distributing and analysing surveys, and interviewing key individuals. In the second phase we analysed food- and household waste data in order to decide how to divide the area into a control- and a treatment group (for more information on the pilot study and a list of the barriers uncovered see Appendix A in Supplementary Material). Once barriers had been identified we conducted a non-systematic literature review with the aim to identify suitable tools for addressing those particular barriers.
3. **Designing the Implementation**
  The barriers and behavioural insight tools identified in step 2 lay the foundation for the design of the intervention. For our purposes a three-page long information leaflet was constructed. The front page of the leaflet was designed to address two barriers uncovered in the pilot study: (1) lack of information (the information about the new station might not have reached out to all residents) and (2) the tenants struggled to tell the difference between the two sorting stations. To address these two barriers the front page featured a picture of a food waste sorting station from the area. Also, the leaflet itself addressed the lack-of-information barrier.
  The subtitle of the information leaflet was using a local descriptive social norm, to encourage recycling, using the phrasing (translated) “*Join your neighbours on Hovmästargatan, recycle your food waste”*^3^, See Figure 1. As mentioned above such messages have shown to have great potential of promoting pro-environmental behaviour in numerous studies, and the same approach have been successfully been used in behavioural economics e.g., in order to nudge people toward tax compliance (Hallsworth et al., 2014).
  Another example of a behavioural insight tool used in the leaflet was phrases designed to be vivid, tangible, and relatable, for example by using formulations such as (translated);
  “*If all households in Hökarängen would sort their food waste it would be enough biofuel to support 15 garbage trucks for a year” “A bus can drive 2.5 km on only one bag of food waste”, “Every Swede produces on average 100 kilos of food waste per year*”.
  Presenting information in a vivid and tangible way increases the likelihood that a message will be attended to initially and more likely to be remembered (Gonzales et al., 1988; McKenzie-Mohr, 2013).
  The attitudes uncovered in the pilot study were pro-recycling, and this was highlighted in the leaflet as follows (translated): “*In a survey recently sent out to households in Hökarängen around 8 out of 10 residents stated that they considered recycling food waste to be “very important*” Aligning the community injunctive norms such as “People in Hökarängen believe recycling food waste is the right thing to do” with the descriptive norms “Join your neighbours […] sort your food waste” could be a persuasive way to frame information (Schultz et al., 2007).
  Moreover to address the barriers “Sorting food waste is an inconvenience”, “Laziness” and “The need for the brown compostable bags” two recyclable garbage bags needed to start recycling was included in the envelope. See Appendix B in Supplementary Material for the full information leaflet and Linder (2016) for the design process in more detail.
4. **Executing the Implementation (On a Smaller Scale)**
  In order to avoid expensive failures or unexpected results, like promoting unwanted behaviour or backlash effects, a smaller scale implementation should be evaluated before broadly implementing the strategy. This also provides an opportunity to modify and adapt the implementation if necessary before the large scale implementation (McKenzie-Mohr, 2013). We designed our field experiment for exactly this purpose. The leaflet was sent out to 264 households in the research area in Hökarängen (see experimental design).
5. **Evaluating the Effectiveness of the Implementation**
  To evaluate the effectiveness of the intervention, a solid experimental evaluation design with one or more “treatment” groups and a comparable control group is strongly advised (Steg and Vlek, 2009). Furthermore, measuring actual behaviour changes over self-reported behaviour changes or intentions is preferable (McKenzie-Mohr, 2013; Clayton et al., 2016). Self-reports have been shown to not always correlate well enough with observed behaviour (See e.g., Corral-Verdugo, 1997). We analysed our field experiment using a difference-in-difference method (see below), with the outcome variable being the amount of food waste and household waste collected from sorting stations in the research area.

### Experimental design

To evaluate the effect of the leaflet a NFE was designed. A NFE is an experiment in a field environment where the subjects are unaware of being part of the experiment (Harrison and List, 2004). To address the ethical concerns of NFE, and potentially creating real world effects with the implementation (Cohen, 2013), every contact with residents was approved by Stockholmshem, furthermore in an effort to avoid any manipulative aspects the purpose of the leaflet was clearly stated on the front page, in line with Hansen and Jespersen (2013)

**Figure 2:**
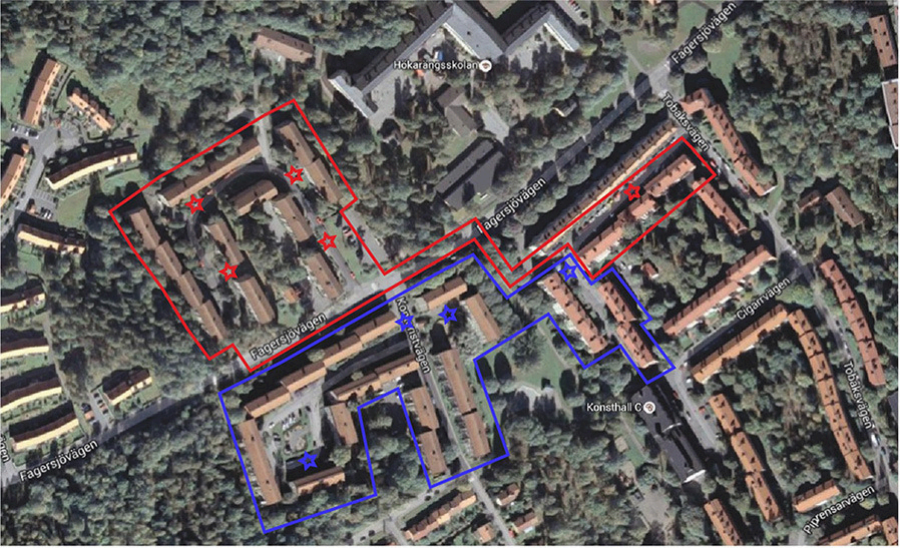
Satellite picture of the research area. The blue area represents the treatment group, all the houses within that area got the information leaflet delivered to them. The red area represents the control group which got no information. The red and blue stars show where the sorting stations are located. Source: Google, Kartdata.

In total 474 households were targeted in the study, with 264 households in the treatment group and 210 households in the control group. The two groups were divided geographically by their blocks to avoid potential spill-over effects. Nine sorting stations were located in the research area, five in the control group and four in the treatment group (see Figure 2). We assumed that the households would use the closest sorting station. Note in Figure 2 that a few households from the control group (red) are in close proximity to both a red and a blue sorting station. In these cases we assumed that they would tend to choose the sorting station located on the way to the centrum, subway and grocery store more often. These households were therefore placed in the control group.

#### Evaluating the implementation

To analyse the results a Difference in difference (DiD) analysis was used. DiD is one of the most popular tools in applied economics research for evaluating the effects of public interventions and other treatments (Abadie, 2005). DiD has, for example, been used to evaluate the effectiveness of policy implementation (see e.g., Finkelstein, 2002), the effect of nudges in field experiments (Kallbekken and Sælen, 2013), and the impact of natural disasters (Tian and Guan, 2015). DiD is especially useful when the aim is to estimate causal effect of an implementation and there is no way to randomly select the population in the control and treatment group which was the case here; the control and treatment groups were defined by geographical conditions. DiD is designed to control both for pre-treatment differences between the treatment and the control group and for trends over time that are unrelated to the intervention (Gertler et al., 2016). The most basic DiD design analyses data for two groups (treatment and control group) over two time periods (pre- and post-implementation). In this study we have one treatment and one control group but several time periods. The treatment group was exposed to the treatment, and the control group was not. The average gain in the control group can then be subtracted from the average gain in the treatment group to calculate the Average treatment effect (ATE).

DiD is thus a suitable method to analyse the effectiveness of the information leaflet, but only if some key assumptions hold true. For one, the parallel trend assumption; that is in the absence of the treatment, the average outcomes for the treated and control groups would have followed parallel paths over time (Gertler et al., 2016). Moreover, we assume that no spill-over effects occurred; that the implementation in the treatment group did not affect the households in the control group. The analysis also assumes a constant group composition over time, i.e., that the type and size of households in the two groups remained constant over the time period. These assumptions will be revisited in the discussion.

## Results

### Data

**Figure 3:**
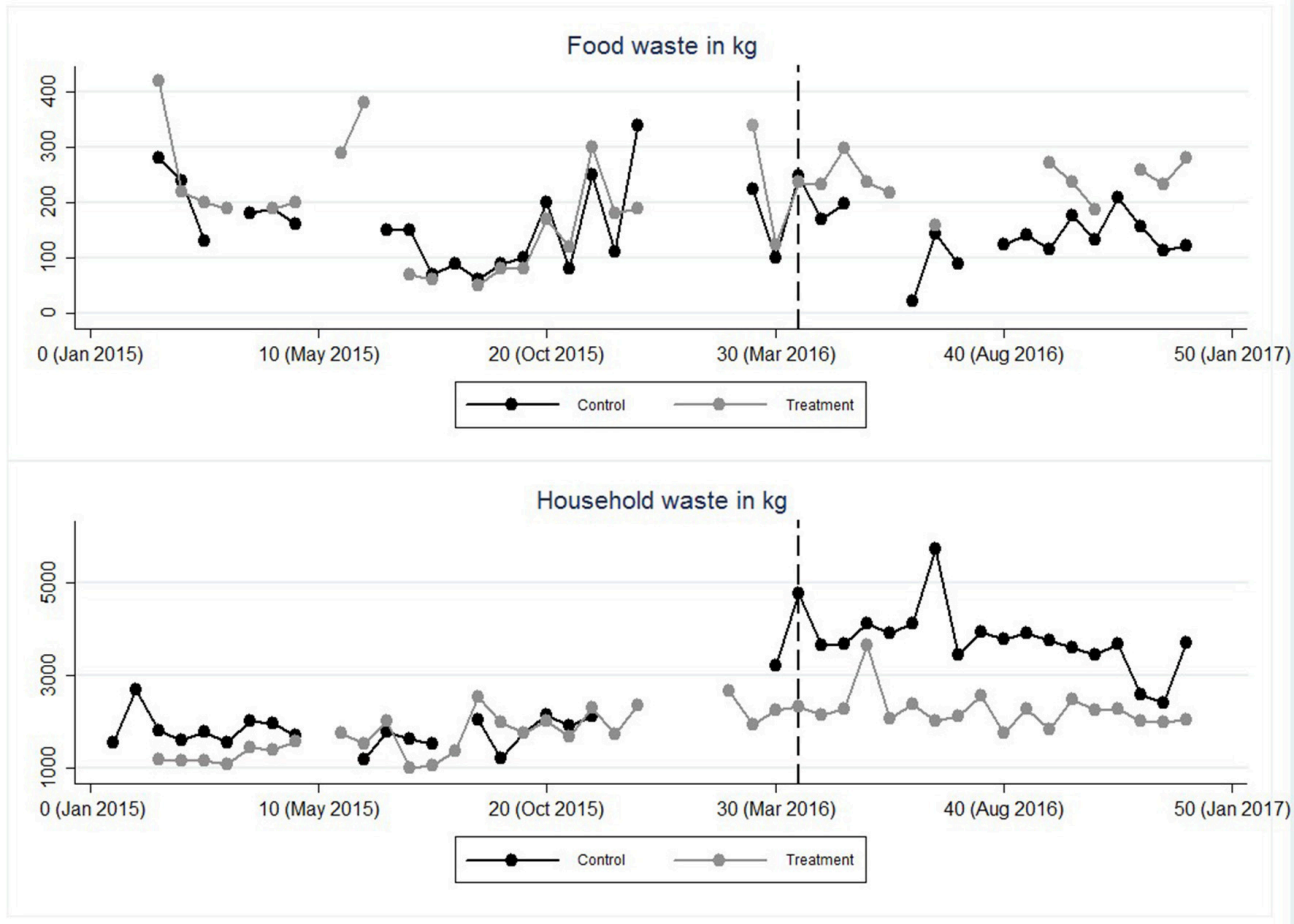
Data points indicate aggregated data on food and household waste registered for all stations in the treatment and control group respectively. Each point represents waste collected over the 2-week period described above starting from February 2015 to December 2016. Note that some of the variation is due to different number of collections in each period. Data points with more than one missing value is removed from the plotted data (but included in the regression analysis).

Our data set include data on food and household waste gathered from nine sorting stations in the research area from January 1st 2015 until December 31st 2016, the waste was weighed and reported by the waste collection vehicles during each collection. Food waste was collected and reported (in kilos) every second week on average, and in total we have data from 373 collections stretching over the entire period. Household waste was collected more frequently (756 collections), but for comparison we aggregate these collections to match the food waste data into 48 different time periods (see Figure 3). In total 13,211 kilos of food waste and 194,736 kilos household waste was collected from the research area. There were several occasions when no record of collection was reported for a certain time period, or waste collection was reported but no weight was recorded (spread over the nine sorting stations) these are registered as missing values. In total there were 95 missing values on food waste, and 43 missing values on household waste. Furthermore, we decided to remove the first reported collection of newly installed food waste stations as we cannot know for sure how long it took before this first collection after installation. Those weights could therefore be misleading and not comparable to the other time periods, we removed in total 19 observations, in total the dataset consisted of 665 aggregated data points of which 302 was food waste data.

### An overview of the results

Figure 3 shows an overview of the total amount of household- and food waste collected before and after the information leaflet was sent out (indicated with the vertical dashed line).

The leaflet was sent out to all apartments in the treatment group on April 14, 2016. Visually it looks like a treatment effect might have occurred (both for the food waste and the household waste). To explore the results further, Table 2 lists the average amount of food waste each station collected in the time period before the treatment, and after the treatment, and the difference between the two groups.

**Table 2:** Average amount of food waste and household waste collected per sorting station in the control group (five stations) and in the treatment group (four stations) before and after implementation and the difference between the groups before and after implementation.

	**Food waste Pre-intervention (T1) (kg, average per station)**	**Food waste Post-intervention (T2) (kg, average per station)**	**Difference (T2–T1)**
Control (CG)	37.67 (29.76)	27.81 (13.67)	−9.86
Treatment (TG)	57.31 (55.67)	59.77 (25.04)	2.46
Difference (TG-CG)	19.64	31.96	
			*DiD (ATE)12.32*
	**Household waste Pre- intervention (T1) (kg, average per station)**	**Household waste Post-intervention (T2) (kg, average per station)**	**Difference (T2–T1)**
Control group (CG)	419.03 (196.95)	744.32 (190.49)	312.6
Treatment group (TG)	472.45 (167.15)	559.31 (152.92)	99.7
Difference (TG-CG)	53.42	−185.01	
			*DiD (ATE) −212.9*

*Standard deviations in brackets. The Difference-in-difference (DiD), or the average treatment effect (ATE) is presented in italics*.

Table 2 shows that the difference in the average amount of food waste collected between the two groups was higher after the intervention; before the intervention the average amount of collected food waste in the treatment group was 19.64 kg more than the control group (57.31 kg compared to 37.67 kg), and after the intervention the difference between the control and the treatment group was 31.96 kg. The average amount of household waste collected was 53.42 kg more in the treatment group compared to the control group before the leaflet. After the leaflet was sent out an average of 185.01 kg less household waste was collected in the treatment group compared to the control group. These numbers then suggest that the estimated ATE for food waste was positive and amounted to 12.32 kg collected per station, every 2 weeks (corresponds to an increase of about 26% compared to a pre-intervention average). The estimated ATE for household waste was negative and equal to −212.9 kg collected per station every 2 weeks (corresponds to a decrease of about 48% compared to a pre-intervention average).

**Figure 4:**
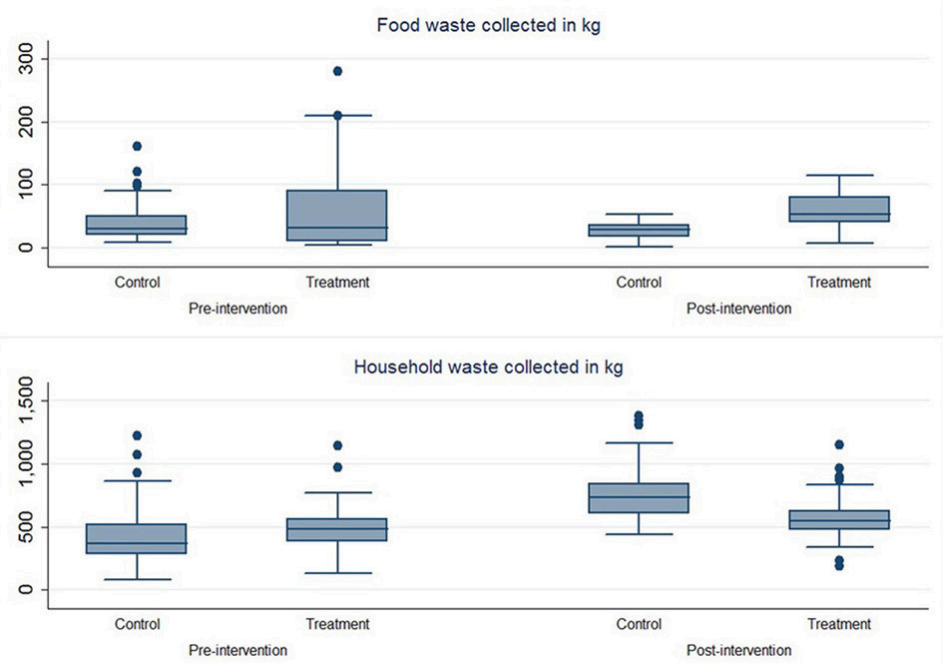
Box plots of the amount of food waste collected (upper) and household waste collected (lower) in the control group and the treatment group pre- and post-intervention.

The positive ATE for food waste, and the negative ATE for household waste can also be illustrated in Box plots (see Figure 4). Looking at the median of food waste collected before and after the treatment we can clearly see an increase in the treatment group and a relative unchanged control group. The box plot also highlights the fact that there was an increase of household waste collected in both groups, but that the increase was considerably higher in the control group. Figure 4 might be more representative (compared to Table 2) for the treatment effect on food waste, because of some outlier values in the beginning of year 2015 (a year before the intervention) driving up the pre-intervention average in the treatment group (we chose not to remove any outliers in our data set), this means that the ATE, looking at averages, might be underrepresenting the actual effect of the intervention. The overview of data still indicates that the intervention had the desired effect. To see if these results are statistically significant we performed a regression analysis.

### Regression analysis

A multivariate linear panel regression was executed using STATA 12, which allowed us to control for other variables that may have influenced the results (such as the number of collections in each time period). Results from the regression analysis can be found in Table 3 below. A fixed effect model was used (as opposed to a random effects panel data model) to account for the fact that the population was not randomly selected (Baltagi, 2008). To account for potential serial correlation we used robust standard errors clustered at the group (station ID) level (Bertrand et al., 2004).

**Table 3:** Average treatment effects for food waste and household waste through a panel data regression model, robust standard error in brackets.

	**Food waste**		**Household waste**	
	**Coefficient (Robust St. error)**	***p-value***	**Coefficient (Robust St. error)**	***p-value***
Constant	15.76 (9.62)	0.140	−37.72 (30.55)	0.252
ATE	13.06^*^ (3.98)	0.011	−221.54^*^ (66.32)	0.010
# Collections	22.70^*^ (7.58)	0.017	237.49^**^ (10.461)	0.000
Post Intervention	−4.89^†^ (2.48)	0.084	287.52^**^ (35.14)	0.000
Model test	6.59^*^	0.0149	197.51^**^	0.000
N	296		364	

We let

† *denote significance below 0.1*,

* a significance below 0.05 and

** *a significance below 0.01*.

The regression analysis confirmed that the ATE of the intervention was significant for both food (*p* < 0.05) and household (*p* < 0.05) waste. The regression analysis revealed that the leaflet had an ATE of 13.02 kg of food waste per station per time period (which equals about 52 kilos more food waste collected every other week). We can also see in Table 3 that the intervention had a negative significant ATE on household waste of 221.54 kg per station. Note that these numbers are similar to the ones estimated above, but here accounts for the number of collections and potential serial correlation within stations.

Because we used kg/collection as dependent variable in the regression analysis we also controlled for the number of collections in each time period. The number of collections in each period (how many times the garbage truck collected waste in the given time period) had a significant impact on the waste collected per station in each time period. In the DiD regression we also needed to control for potential external effects that may have affected behaviour in both the treatment and the control group at the same time as the actual intervention was implemented; what if it suddenly was warmer outside, or there was a policy of some sort that affected waste behaviour? Such potential effects were controlled for by the variable “post-intervention,” which is a dummy variable for post intervention observations. Without such a dummy we would run the risk of overestimating the effect of the intervention. The model test (an F-test) which compares a model with no predictors to the specified model shows significance.

In summary, we cannot reject our main hypothesis: the information leaflet increased the amount of food waste collected from the sorting stations in the treatment group compared to the control group. Moreover, these results are also aligning with our secondary hypotheses: The information leaflet decreased the amount of household waste collected from the sorting stations in the treatment group compared to the control group.

Next we looked into behavioural patterns over time. Visually it looks like the effect persists over time (see Figure 3). Ideally, to test for patterns over time one would run a regression with additional interaction variables testing for lag-effects of the intervention. One would then compare the effect of the intervention at the time of the actual intervention (the ATE) with the coefficients of the additional lag-periods. Lag coefficients of the same sign and of similar magnitude would indicate that the effect of the intervention remained stable. Larger lag coefficients for food waste (and vice versa lower for household waste), would mean that the effect of the intervention increased over time and smaller lag coefficients that the effect of the intervention attenuated over time. Unfortunately, we did not have enough observations (per period) to include several potential lag-periods. Instead, we ran several regressions where we in each regression tested for one potential lag effect. The estimated coefficient (the ATE) of the actual intervention period is labelled β_0_ in Table 4 below^4^. We tested if the effect of the intervention lasted after 2, 4, 6, 8, and 10 time periods post intervention (with estimated coefficients β_2_, β_4_, β_6_, β_8_*, and* β_10_).

**Table 4:** Behavioural pattern over time for food and household waste.

	**Lag period 2**	**Lag period 4**	**Lag period 6**	**Lag period 8**	**Lag period 10**
**FOOD WASTE**					
ATE	*β_0_*	*β_0_*	*β_0_*	*β_0_*	*β_0_*
	9.07	12.95^*^	–	16.69^**^	16.25^**^
Lag effect	*β_2_*	*β_4_*	*β_6_*	*β_8_*	*β_10_*
	7.40^*^	5.69	–	7,42^†^	6.92
Model test, *F*_(4, 8)_	4.15^*^	16.51^**^	–	6.95^*^	7.60^**^
**HOUSEHOLD WASTE**					
ATE	*β_0_*	*β_0_*	*β_0_*	*β_0_*	*β_0_*
	−109.96^*^	−133.27^*^	–	−220.98^*^	−288.37^**^
Lag effect	*β_2_*	*β_4_*	*β_6_*	*β_8_*	*β_10_*
	57.85	48.89	–	52.93	38.74
Model test, *F*_(4, 8)_	63.90^**^	58.03^**^	–	87.60^**^	98.40^**^

The coefficients have been estimated through a fixed effects panel data regression model, using robust standard errors clustered at the station level. We let

† *denote significance below 0.1*,

* a significance below 0.05 and

** *a significance below 0.01. The coefficients for lag period 6 could not be estimated because of multicollinearity*.

Looking at the food waste estimations in Table 4 there seems to have been a slight lag effect in the first estimation; when we tested the effect of the intervention after 2 periods (about 4 weeks) we saw that indeed there seemed to be a slight delay in the effect of the intervention. Table 4 shows that all other ATE coefficients are positive and significant for food waste. Similarly are all coefficients for household waste negative and significant. Please note that the coefficients for lag period 6 could not be estimated because of multicollinearity. Table 4 thus confirms that a significant behaviour shift occurred at the time of (or shortly after) the actual intervention. Unfortunately, it is hard to make additional inferences about behavioural patterns over time based on these estimations. All estimated lag coefficients are insignificant (except for one showing a positive lag effect for food waste even 8 periods - about 4 months - after the intervention). The only conclusions we can draw with certainty is that the effect of the intervention lasted even 8 months after the intervention regardless of any potential attenuation over time.

### A robustness check

Most of the missing values occurred in the beginning of 2016 when a new waste collection company was contracted. The transition did not go smoothly and as a consequence no waste collection data could be recorded for the first two and a half months of 2016 (time periods 25–28). It is important to note that this affected both the control and the treatment group and occurred before we implemented the experiment. Nevertheless, we wanted to control for a potential pre-treatment (lead) effect in a regression where we included a “fake” treatment period; the weeks after the problems were sorted out but before our implementation. For food waste data we found no such effect (the *p*-value of the coefficient equals 0.829). For the household data however the results indicate that something did happen before the intervention, but in the opposite direction (to the ATE). Household waste *increased* just before the intervention, (*p*-value < 0.001) but then decreased post intervention (this trend can be observed in Figure 3). The regressions can be found in the Appendix C (Supplementary Material).

## Discussion

The results show statistically significant support that the information leaflet increased the recycling of food waste in the area. The difference between the control and the treatment group persists and is significant even 8 months after the information was handed out. Before discussing the relevance of these findings there is a need for a critical reflection about their reliability.

As mentioned above, the study design and execution was interrupted by the change of waste managing companies, this change affected both the treatment and the control group and resulted in some missing data. Missing data makes it harder to justify the assumption of equal trends in the DiD. Even though the trends look similar before the implementation, the DiD analysis attributes any trend change that might have happen to the intervention. If there are any other confounding variables present that may have changed the trends, the estimation will be biased (Gertler et al., 2016). There is no way to guarantee that these trends remained parallel during the time of the missing data, but the robustness test presented above does support the parallel trend assumption, at least for the food waste data (our main variable of interest), increasing the likelihood that the change observed was caused by the leaflet. The robustness check also supports the assumption that the change in waste collection company affected the treatment group and the control group equally for food waste. Furthermore, to strengthen the assumption in our DiD analysis of a constant group composition, we looked into official statistics (from Stockholm municipality) on Hökarängen for the year 2016. Household sizes stayed essentially the same over the year, with a net population increase of 23 people (to 9,434), and only about 14% of the households changed residents. Since our research area constitute about 10% of the Hökarängen suburb, we think it is unlikely that these changes are big enough to significantly influence our results.

NFEs have inherent weaknesses and there is always a risk for unknown factors affecting the outcome (Harrison and List, 2004). Our analysis shows evidence of confounding factors influencing the household waste data that manifests itself in the lead effect presented above. This makes it impossible to fully attribute the ATE to the intervention for the decrease of household waste collected. The trend responsible for the lead effect shows a rapid increase in household waste just before the intervention, this trend is visible in both groups but appears to be stronger in the control group (See Figure 3). Fortunately, there is no such lead effect visible in the food waste data. Potential confounding variables are more likely to affect household waste data simply because of the variety of different types of waste being dispatched there. Furthermore, these sorting stations are not locked (the food waste station are locked, and only residents have the key), and in the pilot study we found some reports of “external” households throwing waste into these sorting stations. Due to the limited data available to us we can only speculate about potential causes for this spike, it could be due to the new collection vehicles, or waste coming from external households (or something entirely different). It is therefore important to highlight here that the reliability of the statistical significant results in the household data is low. That being said, the hypothesized trend seems to continue after the lead effect occur and combined with the significant results in the food waste data (which show no sign of such confounding variables) it is still likely that some of the change observed in the household data is caused from sorting out and recycling food waste.

As stated above there is a lag effect in the interventions effect on food waste, however, this is to be expected since it will take some time from the point residents decided to starting sorting food waste, filling up the bag and recycling, before the weight gets registered by a collection vehicle.

The concerns and potential biases mentioned above should be taken into account when evaluating the results from this study. Nevertheless, the results do indicate that by using a theoretically informed design process—an information leaflet using psychological insights can have a significant effect. While at the same time being easy and cheap to adapt, scale up and repeat. There is often an economic incentive for housing companies to promote the recycling of food waste amongst their residents. Stockholmshem for example, paid 610 SEK (64 Euros) per ton for the collection of household waste and 350 SEK (37 Euros) per ton for food waste at the time of the study. Considering the low costs involved in the execution of this study, and the promising results, it seems the leaflet is both an environmentally and economically sound strategy for housing companies, presenting a possible win-win situation. And even more promising since the difference between the control and the treatment group seems to persist even 8 months after the intervention. Our study provides some further support for the use of methodological frameworks such as the ones presented by CBSM and Steg and Vlek (2009). The next natural step for Stockholmshem would be a larger scale implementation of the intervention in Hökarängen, adding to the methodological framework used in this study this would be step *(6) Large scale implementation*, only after testing and evaluating at a smaller scale, a large scale implementation of interventions is advised, which should if possible also be evaluated (Steg and Vlek, 2009).

The encouraging results motivate further research. Relevant next steps would be to test different follow-up implementations (e.g., reminders, prompts or other complementary measures) in order to secure stronger long-term effects, and test the methodological framework on other behaviours and in other contexts. Since we only measure the behavioural impacts of the information leaflet, future research could follow up with qualitative approaches exploring how interventions like this one affect attitudes and values and how these may change over time, as well as exploring potential spill-over or reactance effects. For example it is possible that the households that started recycling food waste will be more willing to throw out food since they perceive it to be put to good use anyway hence increasing their waste production instead of only recycling waste that would otherwise been unsorted household waste. On the other hand it is also possible that food waste recycling can have a positive spill over on other pro-environmental behaviour, a recent study provided some proof of just that (Sintov et al., 2017). Since this study is focusing on evaluating and testing the intervention as a whole and not a specific behavioural insight tool, we cannot assert with certainty which tool worked or contributed more than another. Qualitative approaches, coupled with experiments with the particular purpose to distinguish between tools could shed further light on these questions. Due to the complexity of human behaviour we want to re-emphasize the importance of testing and adapting interventions to the specific context and target group. Some of the barriers found in the pilot study are highly contextual, and demographical traits such as political support for the socialistic block and pro-environmental norms in Hökarängen might have been strong contributing factors to the seeming success, such demographics have previously shown to influence how susceptible people are to pro-environmental interventions (Costa and Kahn, 2013).

The design of the information leaflet and the research approach of this study rest on combining insights, terms and theoretical frameworks from both environmental psychology and behavioural economics, in particular linking CBSM and nudging. It is apparent that a lot of promising research on how to promote pro-environmental behaviour is occurring both within behavioural economics and environmental psychology today, but surprisingly little cross references occur. There has been some expressed concerns about how to integrate these frameworks (Badshah, 2010). However, based on the experiences gained during this study no obvious obstacles in combining the frameworks were encountered, we had rather the opposite experience. CBSM generally has a more holistic view on behaviour change and present guidelines in line with the ones used in this study. The rapidly expanding field of nudging, based on insights from lab and field experiments could be an important contribution when designing interventions, and adding insights from the growing field of nudging, as a way to expand the toolbox for changing behaviour is therefore something that we recommend for CBSM-researchers. On the other hand, practitioners from behavioural economics and nudging could equally learn from social marketing approaches and environmental psychology, especially when it comes to the strategic planning process e.g., how to find and address barriers, or how to select which behaviour to change. We would recommend researchers taking a nudge approach to follow a methodological framework similar to the one used in this study. As mentioned before nudging approaches have been criticized for being insufficient responses to current environmental challenges, and similarly Corner and Randall (2011) state that “Social Marketing alone is insufficient to build support for the more ambitious policy changes and interventions that constitute a proportional response to climate change” (p.1). We also want to emphasize that interventions using psychological insights to spark behaviour changes amongst individuals should not be viewed as a silver bullet solution to our current sustainability problems. Only focusing on promoting pro-environmental behaviour amongst individuals would not suffice to create the large scale transformation needed. For example, it is estimated that about 44% of food waste in the US is stemming from households, but the rest is from manufacturing, retail and food service sectors (Vogliano and Brown, 2016). Thus behaviour change needs to be integrated and operationalized across sectors, organizations, policy, as well as among individuals.

Nevertheless, designing more effective interventions looking at quick achievable behaviour changes with the potential of big aggregate impacts without reducing human well-being is arguably a good way to complement other measures. Insights gained from this study and behavioural science are useful not the least when designing interventions in cities. Urban areas are complex systems, often rich in terms of cultural diversity, world-views and life-styles co-existing on dense geographical scales (Colding and Barthel, 2013). Designing and building new urban landscapes for 3.2 billion new inhabitants before year 2050 without generating devastating environmental and social impacts is a grand challenge indeed. However, we see opportunity in the fact that the world moves toward a more densely settled urban population (UN, 2015) as it allows for interventions in contexts and action arenas where small scale changes can reach many people and therefore have large aggregated effects. These are environments in which interventions using psychological insights could prove to be especially effective tools to promote pro-environmental behaviour.

## Ethics statement

This study was carried out in accordance with the recommendations of APAs Ethical Principles of Psychologists and Code of Conduct. To fulfil the requirements stipulated in (Swedish) Act concerning the Ethical Review of research involving humans (2003:460) we did not collect any sensitive data. The data collected was purely aggregated waste data from sorting station in the research area, with no way of connecting it to individual households or individual behaviour. Every contact or message sent to residents in the research area was approved and consent given by Stockholmshem, and the purpose of the information hand-out was clearly stated on the front page (see Figure 1) in line with, Hansen and Jespersen (2013). The pilot study presented in this study was conducted as a part of Linder's (2016) master thesis and was aligning with ethical guidelines for master thesis works at Stockholm Resilience Centre, an ethical statement was written and approved by the centre before the study started. To fulfill the requirements of the Swedish Personal Data Act (1998:204), each subject was informed about the purpose of the pilot, how the information would be used and their anonymity was maintained. They could remain anonymous at all times if they preferred.

## Author contributions

NL, TL, and SB all contributed to the initial idea, the design and planning of the study. NL collected the data, conducted the pilot study and designed the intervention with inputs from TL and SB. Designed the field experiment with inputs from TL. Wrote the majority of the text and handled the collaboration and communication with Stockholmshem. TL led the data analysis with input from NL, wrote most of the results section. Made the graphs and tables. Contributed to the writing and provided continuous feedback on the whole manuscript. SB initiated the collaboration and contacts which made the study possible, contributed to the writing process, and provided continuous feedback on the whole manuscript.

### Conflict of interest statement

The authors declare that the research was conducted in the absence of any commercial or financial relationships that could be construed as a potential conflict of interest.

## Footnotes

^1^For example the “Behavioural Insight Team” in the UK, similar governmental initiatives are seen in the US, Canada, Australia and New Zealand. In Norway and Denmark non-profit organizations for nudging have been initiated (Mont et al., 2014), as well as the European Nudge Network (http://www.behaviouralinsights.co.uk; http://tenudge.eu/).

^2^For a more in-depth guide to each of the steps see Steg and Vlek (2009), McKenzie-Mohr (2013) and Linder (2016).

^3^“Hovmästargatan” is the name of the street the participants live on.

^4^The actual regressions can be obtained upon request from the authors.
