# Supplementary material for: Using Behavioural Insights to Promote Food Waste Recycling in Urban Households—Evidence From a Longitudinal Field Experiment
Source: Front Psychol. 2018 Mar 22;9:352. doi: 10.3389/fpsyg.2018.00352 (PMC5875413; doi:10.3389/fpsyg.2018.00352)
Supplement: Supplementary file 1 [file DataSheet1.docx]

# Appendix A

A request to participate in a survey was distributed to 92 households (around 20%) in the research area. The households targeted for the survey were evenly distributed among the treatment group and the control group. Each apartment complex in the two groups was given a number and then selected using a random number generator and flipping a coin (each house had two entrances and the coin flip decided in which entrance the request would be handed out in). The targeted households were provided with a short information about food waste and asked to answer 9 short questions about their household’s food and household waste habits, after about 2 weeks a reminder was handed out (13 households responded to the survey).

To complement the survey, two semi-structure interviews were conducted. One interview was with a worker at Stockholmshem (with responsibility for the area), and one interview with a local resident from the area with over 40 years of living there and with good knowledge of both how the area works and how Stockholmshem is operating in the area. This contact was provided through Stockholmshem. Note that the findings from the pilot study is not meant as scientific results, merely to provide guidelines for the development of the intervention.

The pilot study uncovered the following potential barriers for recycling food waste, see Table 5 below:

| *Table 5. The barriers uncovered in the Pilot study, their sources and the reasons for being perceived as a barrier. The barriers are listed in order with the perceived biggest barriers listed first.* | | |
| --- | --- | --- |
| **Barrier** | **Source of barrier** | **Reasons for being perceived as a barrier** |
| Lack of information, the information did not reach everyone | Interviews, Survey | Both interviewees stated a major concern that they thought the information about the new sorting stations did not reach out to everyone.  This worry was supported by an answer in the survey. One respondent stated that the reason he didn’t sort food waste was because it was too far to the sorting station, and he had to take the car (the longest possible way to a sorting station is about 200 meters). |
| Can’t tell the difference between the sorting stations. | Interviews | The sorting station for food waste is very similar to the station for household waste. It might not be obvious to everyone what the difference between them are, especially if the information did not reach everyone. This confusion also manifested itself in the fact that the wrong type of trash, mostly plastic bags and household waste, was thrown in the sorting station for food waste and contaminated the waste. |
| The belief that the garbage truck doesn’t sort the waste anyway. | Interviews | There is a belief in the area that the garbage truck doesn’t sort the waste, that they dump it all in the same truck, and thus that all the sorting in households are obsolete. This is raised as a concern in both interviews “The local” brought this up as well. He believed that the garbage men did that sometimes out of laziness. He also stated that several others in the community have seen it happen. “The Stockholmshem worker” said that this was indeed happening (the truck sometimes sort everything in the same truck), but it’s not due to laziness, it’s when plastic bags and other garbage gets thrown into the food waste stations, contaminating the food waste and thus needs to be sorted as regular household waste |
| Sorting food waste is an inconvenience. | Survey | Inconvenience reasons mentioned in the survey:  “Not enough space for another garbage bag”,  “Living alone means very small amount of food waste, need to keep track of having compostable bags”.  “I walk with a crutch, so I can only carry one bag at the time” |
| Laziness. | Survey | Laziness was stated as one of the reasons for not sorting food waste. |
| The need for the brown compostable bags | Interviews, Survey | To start recycling food waste residents need to acquire compostable bags. These are available for free in the close-by waste room, but it’s not certain that everyone knows that, and they also need to remember getting one to be able to start recycling food waste. |
| Cultural differences. | Interviews | Cultural differences were brought up as a potential barrier in the interviews, but the views differed about how big this impact might be. Mostly it came down to not understanding the language, and the information, but normative differences in what’s perceived as normal when handling trash were also mentioned. Although the Stockholmshem worker said that he did not see the need for translating information leaflets. |
| People don’t understand the information | Interviews | There might be a language barrier in the way of understanding the information sent out. |
| The sorting station stinks during summer. | Interviews | In both interviews this was mentioned as a major issue. The food waste sorting stations starts to stink during summer due to food waste going bad. The local profile even told me he heard of people moving just because of the stench. At Stockholmshem they were well aware of the problem, and proclaimed that they now have solved it with more regular cleaning. |
| The stations are hard to open. | Interviews | “The local” had heard some people in the area complaining about the stations, that they are hard to open. A key is needed to open the station (municipal regulation), this provided an obstacle for some of the elderly people since the lock is located pretty far down on some of the stations. |

# Appendix B

The information leaflet sent out to the housholds in the research area is translated from Swedish to English and presented in Figures 5, 6 and 7 bellow. Figure 5. Picture of the front page of the information leaflet. The picture used was taken by one of the authors.


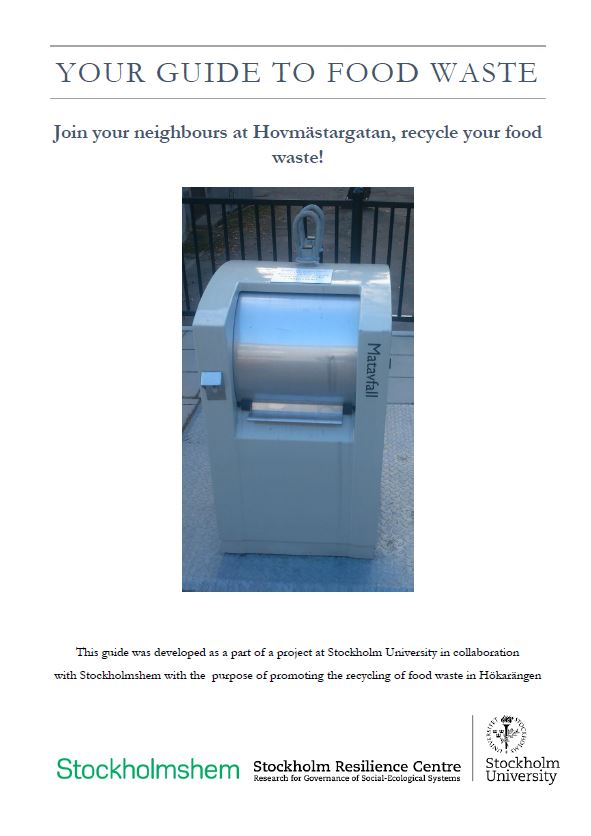


Figure 6. Picture of the second page of the information leaflet, the picture and permission to use it was provided to us by Stockholm Vatten.Figure 7. Picture of the third page of the information leaflet, the picture, and permission to use it provided to us from Stockholm Vatten.


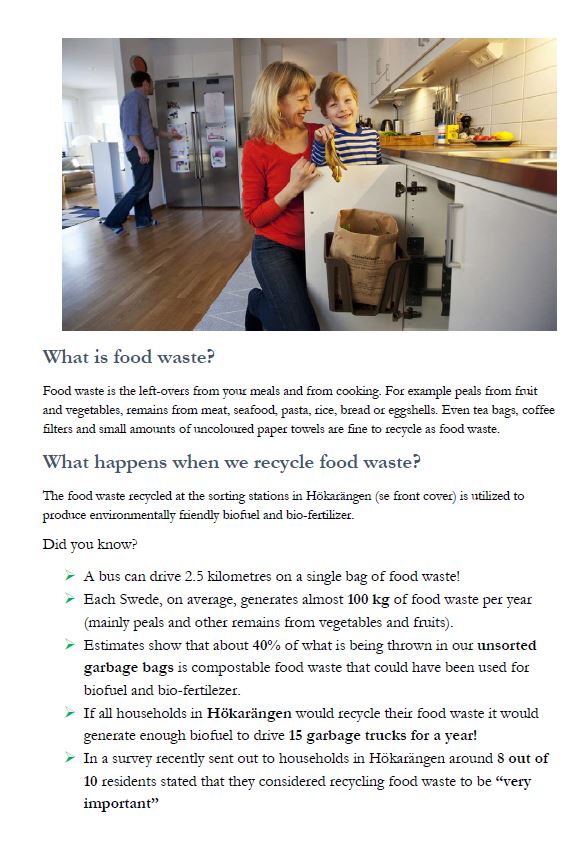

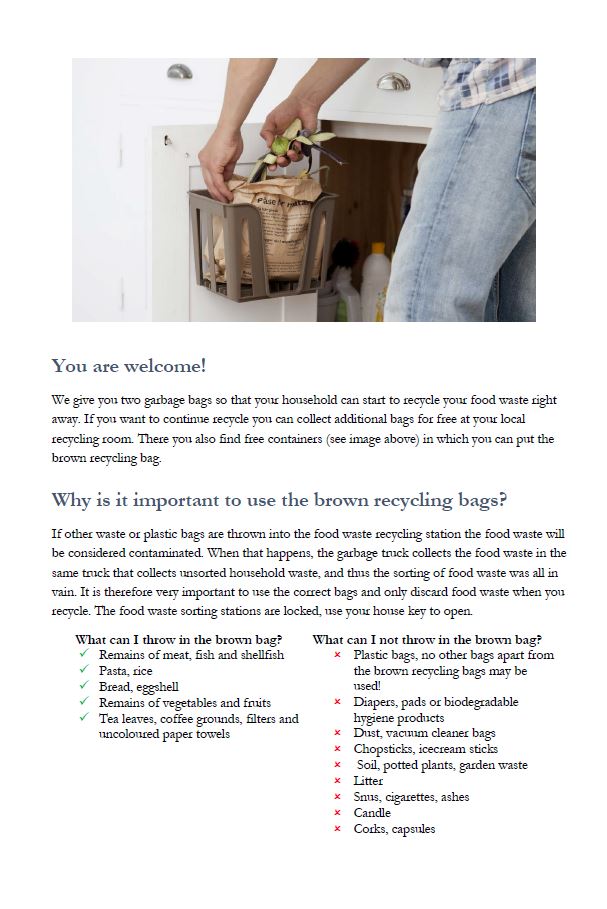


# Appendix C

The robustness check regression is presented in Table 6.

Table 6. Average treatment effects, including the test for a potential lead effects, for food waste and household waste through a panel data regression model, robust standard error in brackets. We let ^†^ denote significance below 0.1, * a significance below 0.05 and ** a significance below 0.01.

|  | **Food waste** | | **Household waste** | |
| --- | --- | --- | --- | --- |
|  | Coefficient  (Robust St. error) | *p- value* | Coefficient  (Robust St. error) | *p-value* |
| Constant | 15.76  (9.67) | 0.142 | - 43.36  (37.16) | 0.277 |
| ATE | 12.93**  (3.68) | 0.008 | -198.22*  (72.04) | 0.025 |
| # collections | 22.74*  (7.69) | 0.018 | 235.12**  (13.29) | 0.000 |
| Lead effect | -0.79  (3.54) | 0.829 | 145.79**  (40.89) | 0.007 |
| Post Intervention indicator | - 4.88**  (2.49) | 0.086 | *288.00***  *(35.24)* | 0.000 |
| Model test | 17.69** | 0.000 | 113.19** | 0.000 |
| N | 296 |  | 364 |  |
